# Supplementary material for: The State of the Evidence about the Family and Community Nurse: A Systematic Review
Source: Int J Environ Res Public Health. 2022 Apr 6;19(7):4382. doi: 10.3390/ijerph19074382 (PMC8998909; doi:10.3390/ijerph19074382)
Supplement: Supplementary file 1 [file ijerph-19-04382-s001.zip › ijerph-1594470-supplementary.pdf]

Table S1. Synthesis of the main characteristics of 90 included articles.

	First author and year of publication	Title	Aim	Study design	Population	Country	Theme	Results
1	Adamson E. (2013)	A "good death" at home: FCNs helping to make it possible	To discuss the results of recent research (Cruickshank et al, 2010), illustrating the central role that FCNs in the choice to die at home	Discussion paper	FCNs	UK	Clinical practice	FCNs play a key role in ensuring the respect and dignity of patients in palliative care, in particular to support patients and their families in the process of accompanying death.
2	Adderley J. (2015)	FCNs' judgement for the management of venous leg ulceration: A judgement analysis	1. To evaluate the accuracy of diagnostic and therapeutic judgments in relation to high compression. 2. To explore the use of available information tools for diagnosis and treatment	Cross sectional study	FCNs	UK	Clinical practice	FNCs are tasked with preventing and treating numerous chronic conditions at home, including leg ulcers. In this field, clinical judgments and decisions they are complex and uncertain and may be affected by a certain variability in the judgment and interpretation of information.
3	Aldridge A. (2014)	The role of the FCN in psoriatic comorbidities interventions	To describe psoriatic comorbidities and its impact on patients and the role of FCN	Discussion paper	Patients affected by psoriasis	UK	Clinical practice and core competencies	The FCN must be able to recognize the signs and symptoms of the disease and promote a healthier lifestyle for patients with psoriasis. This change requires the nurses to have good communication skills and to be persistent. to allow the patient to 'own' the change.
4	Andrews N. (2021)	FCNs' support for patients with fibromyalgia who use cannabis to manage pain	To provide information to FCNs on how cannabis use affects the experience of pain for people living with FM	Discussion paper	Patients affected by fibromyalgia	UK	Clinical practice	People with fibromyalgia have their own experience of pain that does not always find relief in traditional therapies. The use of cannabis is a viable alternative with excellent results for these patients and it is important that FCNs deepen their knowledge in this regard to provide adequate and safe care.
5	Bagnasco A. (2020)	A European e-Delphi study to identify the fundamental skills for the	To identify core competencies of FCN	Qualitative study	FCNs	Europe	Core competencies	From this study, which was born among the partners of the ENhANCE project, 27 core competences are defined that an FNC should have.

		definition of the training path of the FCN						
6	Balestra M. (2019)	Family Nurse Practitioner Scope of Practice Issues When Treating Patients With Mental Health Issues	1. To outline the different scope of practice for nurses working with patients with mental health issues 2. To provide recommendations to help them ensure best practices and patient safety.	Discussion paper	Patients with mental disease	USA	Clinical practice	The article addresses the issue of home management of patients with mental disorders by identifying the necessary skills and underlining the importance of being aware of the limits of one's work. Violations of one's own competences or scope of action can lead to civil liability and disciplinary action.
7	Bidone S. (2021)	Evaluation of self-perceived competence among FCNs who attained the Master's Degree at the University of Eastern Piedmont (2013-2019): An exploratory survey	To evaluate the perceived preparation by nurses who have obtained a master's degree in FCN at the University of Piemonte Orientale	Cross sectional study	FCNs	Italy	Core competencies, Outcomes and Advanced training program	The course is divided into learning units, corresponding to the theoretical and practical requirements of the FNC who has found a high level of skills in individual care, while recording much lower levels for community care, especially in defining short- and long-term outcomes and in promoting improvements in lifestyles.
8	Bright T. (2019)	Smoking cessation and the health promotion role of FCNs	To discuss the health-promoting role of a nursing student during the coaching in a community nursing team	A case study	A smoker patient	UK	Clinical practice and Core competencies	The literature shows that PHC is the primary area where FCN feels best represented in its key role, including the goal of smoking cessation. Understanding the patient's point of view is essential to effectively involve them in the care process.
9	Broekema S. (2020)	Effects of FCN conversations on families in home health care: A controlled before-and-after study	To assess the effects of FCNs conversations on family caregiver burden, patients' quality of life, family functioning and the amount of professional home health car	A quasi-experimental study	Patients and their families	Netherland	Outcomes	The study shows that for the group followed by the FCN there was a significant increase in family functioning compared to the control group, while it did not reveal any significant benefits on the patients' quality of life. health care remained unchanged in the control group while it significantly decreased in the intervention group.
10	Cavada L. (2021)	The implementation of family nursing in a municipality	To describe the contents and the learning proposed with specific training and testing of a pilot	Discussion paper	FCNs	Italy	Organizational and educati	Calgary model is a family assessment model and takes into account three dimensions of the family: structural, developmental and functional.

		of the Provence of Bolzano	project on insertion of three family nurses in mountain.				onal models	The assessment of the FCN is guided by questions that evaluate the cognitive, affective and behavioral domain.
11	Chambe rlain V. (2016)	Patients with inflammatory arthritis: an opportunity for FCNs	To give FCNs an overview of the condition, including signs and symptoms, diagnosis and medication while helping to identify ways in which they might be able to become more involved in their direct care.	Discussi on paper	Patients with arthritis	UK	Clinical practice	FCNs should be prepared about the correct use of analgesia and the evaluation of its effects, as well as with conventional and non-conventional pain treatments for patients with arthritis.
12	Chamor ro A. (2014)	The family and FCN: health agent and model for the chronic patient within the community	To describe, analyze and correct the factors that limit professional growth and the supply of a portfolio of FCN services in Spain	Discussi on paper	FCNs	Spain	Core compet encies	FCNs had to implement strategies that promote continuity and quality of care, evaluate the impact of care by defining process indicators and results, have sufficient human and materials resources.
13	Chater A. (2019)	FCN and antibiotic stewardship: the importance of communication and training	To enlighten effects of excessive use of antibiotic therapy and identify the role of FCN	Discussi on paper	A 35-year-old male patient	UK	Clinical practice and Core compet encies	The reduction of antibiotic resistance can be reduced through proper prescription management. The prescribing nurse must guide his or her decisions in terms of capacity, opportunity and motivation. He must therefore have good communication skills and knowledge to deal with patient resistance.
14	Clare C.S. (2017)	The role of FCNs in stroke prevention	To describe stroke epidemiological data, risk factors and prevention interventions highlighting the role that FCNs can have in primary prevention	Discussi on paper	A patient admitte d to hospital after a potentia l TIA	UK	Clinical practice	Health promotion and prevention of acute events, including cerebrovascular events, are among the key activities of FCNs. For this purpose, FCNs can work in multidisciplinary teams with the role of case manager. starting from the understanding of patients' needs, they can develop prevention, counseling and treatment actions.
15	Connoll y M. (2018)	Evaluating an educational programme for dignity care intervention with FCNs in Ireland	To present the results of an evaluation of a palliative care education programme targeted on Dignity Care Intervention	Cross sectional study	Publich health nurses and registere d	Ireland	Advanc ed training progra m	FCNs needed to be trained about end-of-life care. This could help the preservation of the patient's dignity, that could be not underestimated and to let FCNs be capable of caring

			(DCI) Ireland for FCNs		general nurses			patients and their families at home.
16	Courtenay M. (2018)	Community nurse prescribing	To describe the state of the art of prescribing FCNs in the UK	Discussion paper	FCN prescribers	UK	Clinical practice and Advanced training program	FCN skills development enabled the nurse to become a prescriber through a ten-day training program after six months of field experience. However, their number is decreasing, and, to date, there are around 36,000. The main cause seems to be the number and type of drugs that they are allowed to pre-write, which does not allow adequate prescriptions.
17	Cramm J.M. (2017)	Self-management abilities and quality of life among frail community-dwelling individuals: the role of FCNs in the Netherlands	To determine whether Dutch FCNs improve the self-management and quality of life of fragile people in their communities	Logitudinal study	Elderly patients	Netherland	Outcomes	The provision of care by FCN to frail people living in communities in Rotterdam produced a significant advantage in improving the quality of life and clinical outcomes especially in relation to self-management skills, as well as in general behavior.
18	Daly B. (2015)	Promoting good dental health in older people: role of the FCN	To describe the characteristics of oral health in the elderly and supportive interventions to be implemented in residential care facilities	Discussion paper	Older patients	UK	Clinical practice	The literature shows that PHC is the primary area where FCN feels best represented in its key role. Among other challenges, FCNs are also engaged in promoting oral hygiene
19	Davis W.A. (2013)	Determinant and costs of community nursing FCN type 2 diabetes from community based observational study: The Fremantle Diabetes Study	To determine the incidence, predictors and costs of FCN provision to patients wth type 2 diabetes in a large community-based representative study of diabetes in an urban Australian setting	Cross sectional study	Patients with type 2 diabetes enrolled in the Fremantle Diabetes Study (FDS)	Australia	Clinical practice and Outcomes	Chronic diseases, including diabetes mellitus in particular, require a high level of clinical practice and FCN skills, for the treatment of complications of the disease in the elderly. Home care for diabetes has an average national cost of 5% to add to the total Australian direct healthcare costs of diabetes.
20	Dening H.K. (2016)	Exploring the community nurse role in family-centred care for patients with dementia	To describe the epidemiology and characteristics of dementia and interventions that can be implemented byFCNs to support the patient and his family	A case studies	Nurses supporting patients with dementia	UK	Clinical practice	The article reports 3 case studies from which it emerges that the FCN plays a central role in the early recognition of the disease and in providing relationship-based care to both patients and family members

21	Dlamini C.P. (2020)	Developing and Implementing the Family Nurse Practitioner Role in Eswatini: Implications for Education, Practice, and Policy	To illustrate the stages of the process of developing and implementing the role of FCN in Eswatini	Discussion paper	Nurses going to be FCN	Africa	Advanced training program	The Nursing Board and Faculty of Nursing have committed to securing courses for the graduate level and have provided FCNs with the knowledge, skills and teaching and clinical skills necessary for successful FCN practice. The program proposal submitted to the university included the curriculum.
22	Duncan D. (2021)	Role of the FCN in Parkinson’s disease and lower urinary disorders	To report the role of FCN in caring patients with Parkinson and lower urinary disorders	Discussion paper	Patients with Parkinson disease and urinary difficulties	UK	Clinical practice	Among the different symptoms of Parkinson's is urinary incontinence. The FCN has a key role to play in assessing these outcomes and supporting families through teaching pelvic floor exercises and accessing the supply of incontinence products
23	Falavigna G. (2020)	The socio-economic planning of a FCNs programme in mountain areas: A Directional Distance Function approach	To verify the sustainability of a European Union project, funded by the Alpine Space Programme.	Cross sectional study	Municipalities with at least on bad outcome in Piedmont	Italy	Outcomes and Organizational and educational models	Consensus model care places older people at the center of health and social care by allowing them to stay at home as long as possible. It is based on the crucial role of the FCN. in order to determine in which places it is more appropriate to implement the model, altitude has proved to be a statistically significant item in the possibility of access to care and services by the elderly population.
24	Flavell T. (2015)	An overview of eczema management for FCNs	To offer an overview of the main eczematous conditions and related interventions that FCNs can implement to manage them	Cross sectional study	Nurses interested in managing eczema	UK	Clinical practice	FCN plays a key role in health promotion and treatment of chronic conditions, including eczema. FCNs must be able to recognize the different types of eczema and implement specific treatments, involving caregivers and the family.
25	Fu W. (2010)	Development of FCN in Zhejiang Province, China: a report of the driving measures	To describe the development of FCN in Zhejiang	Discussion paper	Zhejiang Community nursing	China	Advanced training program and Core competencies	In China, a 1-year postgraduate course in organized in a specific way: it is composed of 6 months theory, 6 months practical, allowing registered nurses to specialize in FCN. The primary care center carries out all health activities, including disease prevention, treatment, rehabilitation, health

								education and technical assistance to family planning.
26	Gafas González C. (2017)	Community and Family Nursing specialisation program: a reality in Ecuador	To train FCN to take care including the patients and his family respecting their cultures and human rights.	A mixed method study	FCNs	Ecuador	Clinical practice and Core competencies	FNCs provide care based on evidence and best practice, have the knowledge, skills and competencies to tailor the care they provide to individual needs. The main activities of FCNs are assistance, education and health promotion and the management and evaluation of its work. They have to work coordinating with other professionals by promoting relations between social and health services
27	Green S.M. (2014)	Barriers and facilitators to screening for malnutrition by FCNs: a qualitative study	To understand FCNs' perceptions of barriers and facilitators to undertaking nutritional screening: identify factors that enable and prevent FCNs, who provide care for adults in their own home, to understake nutritional screening using a screening tool on patients admitted their care.	Qualitative study	FCNs	Australia	Clinical practice	The literature shows that PHC is the primary area where FCN feels best represented in its key role. FCN are committed, among other things, to the prevention of malnutrition. From the interviews it emerges that lack of time is the main problem that hinders a correct nutritional screening, however the use of the proposed tool "MUST" has received positive reviews, with the proposal to include it in the compulsory training that continues that one.
28	Gregg S.R. (2019)	Try-It-On: preparing family nurse practitioners to use holistic integrative interventions to reduce opioid prescriptions in chronic pain management	To prepare future FNPs to use holistic integrative interventions and effective communication skills to decrease overprescribing of opioids for chronic pain management.	Longitudinal study	FCNs	USA	Clinical practice and Advanced training program	FCN provide tertiary health care, supporting patients, families, and communities in illness management, such as chronic pain. However, the pharmacological approach is predominantly used. The study evaluates the increase in the use of holistic treatments by FCNs after an online preparation course, noting an increase in the possibility of prescribing each new approach, with the exception of massage therapy.
29	Holdoway A. (2019)	What more can FCNs do to manage adult malnutrition	To provide an overview of groups at risk of malnutrition in the community and the effects and clinical	Discussion paper	Adult patients	UK	Clinical practice	Health promotion is one of the key activities of the FCN and includes, among others, the prevention of malnutrition. FCN should discuss appetite,

			consequences of non-identification by defining how FCNs can play a critical role in early identification and care					nutrition and hydration daily with his patients. The article sets out some strategies for managing the problem such as fortifying food, encouraging you to try different eating patterns and to change the consistency of foods in case of difficulty in swallowing, personalization of the choice of food and possibly the use of supplements.
30	Howerton C.R. (2011)	Breast augmentation. Motives and the role of the family nurse practitioner	1. To present epidemiological data on additive mastoplasty interventions and its psychological implications. 2. To suggest ways in which district nurses can ensure adequate support for women who resort or want to resort to this type of surgery.	Discussion paper	Women who undergone breast aumentation surgery	USA	Clinical practice and Core competencies	FCNs are recognized as essential for advising women interested in breast augmentation. Therefore, communication skills are fundamental in this approach to understand what a woman's motivation is by helping her to make the best choice. Knowledge about the intervention and its course are therefore a basic requirement for adequate support.
31	Husband J. (2008)	The evolving role of the FCN specialist in palliative care	Describe the epidemiology and characteristics of palliative care in the UK and interventions that can be implemented by FCNs to support the patient and his family	Discussion paper	FCN specialists in palliative care	UK	Clinical practice	The guarantee of patients' respect and dignity and communication skills emerged as a peculiarity of FCN, which play a key role in palliative care, in particular to support patients and their families in the process of accompanying death.
32	Huy N.V. (2018)	The roles of, activities of, and competencies for, community nursing services in rural Vietnam: Implications for policy decisions.	To explore the roles, activities, and competencies required of FCNs in rural districts of Vietnam.	Qualitative study	12 men and 4 women with work experience in local health systems	Vietnam	Core competencies	Educating and promoting health, managing, monitoring and evaluating health activities as part of the team are the fundamental aspects of FCNs.
33	International Family Nursing Association	Position statement on planetary health and family health	Inform IFNA members, educators, researchers, healthcare professionals of the link between planetary health and family health; Offer	Discussion paper	Patients and their families	International	Clinical practice and Core competencies	Planetary health is important, and FCN should take into account of it in sustainable practices. FCN take into account not only patient but also the relationship with his family member. In addition,

	(INFA) (2020)		direction and support for family nursing education, research, and practice on the topic of the planetary health as it relates to family health.					FCN demonstrates skills to collaborate and work in multidisciplinary teams, leadership skills and systems thinking to ensure the quality of nursing.
34	Ippoliti R. (2018)	The sustainability of a FCNs programme aimed at supporting active ageing in mountain areas	Describe and evaluate the sustainability of the CONSENSO project	Discussion paper	FCNs	Italy	Outcomes and Organizational and educational models	The FCN is a key figure in assisting elderly patients, especially in areas furthest from health services. The CONSENSO project implements the figure of the FCN precisely in these areas. However, in order for the project to be sustainable even without European funds, a cost-benefit study is needed. The study demonstrates how, to implement the FCN without added costs for the SSN, it is necessary that the work of the latter is able to prevent falls with hip fracture, saving money to health care system.
35	Jin L. (2020)	Family nursing with the assistance of network improves clinical outcome and life quality in patients underwent coronary artery bypass grafting: a consolidated standards of reporting trials-compliant randomized controlled trial.	To investigate the effect of family nurse activity with network assistance on clinical outcomes in patients undergoing coronary arterial bypass grafting	A randomized controlled study	Patients underwent coronary artery bypass grafting	China	Clinical practice and Outcomes	The psychological aspect is an important aspect in supporting the health of patients, especially to reduce anxiety and stress. FCN is able to significantly reduce both the incidence of complications and mental health disorders and lung function better than the control group. The effectiveness is greater especially in the medium to long term.
36	Johnson A. (2015)	Analysing the role played by district and FCNs in bereavement support	To explore mourning support as one of the roles of district (DN) and community (CN) nurses.	Discussion paper	District and FCNs	UK	Clinical practice and Advanced training program	FCNs are fully involved in the care of patients and their families, including the bereavement phase. To accompany the daughters in this delicate phase it is necessary that they are adequately trained and know how to recognize the different stages of mourning. Duration

								of post-bereavement care should be tailored to the needs and response of individual families.
37	Johnson A. (2015)	Role of district and FCNs in bereavement care: a qualitative study.	Explore the role of bereavement care from the community or district nurses' perspectives	Qualitative study	District nurses and FCNs	UK	Clinical practice and Outcomes	FCNs are fully involved in the care of patients and their families, including the bereavement phase. From the interviews emerge 5 main themes related to the management of bereavement, first of all the question of time, then the specificity of the case, the interprofessional work, one's role and experience. The results show that if on the one hand there is an awareness of bereavement care, on the other there is a lack of training.
38	Jongudomkarn D. (2014)	Development of a Family Nursing Model for Prevention of Cancer and Other Noncommunicable Diseases through an Appreciative Inquiry	To develop family nursing practice in primary care setting in the Isaan region or Northeastern Thailand and to distil what worked well into a nursing model to guide practice	Qualitative study	FCNs	Thailand	Clinical practice, Outcomes and Organizational and educational models	The model included 3 main thematic areas, namely: implementing a participatory action approach by mobilizing the social capital of families, using the family care process and implementing action strategies within communities. Participants agreed that this process encouraged their critical thinking and creativity by allowing them to solve problems in practice.
39	Kelly A.M. (2019)	What can FCNs do for older adults who experience faecal incontinence?	To describe the epidemiology and characteristics of fecal incontinence and interventions that can be implemented by FCN	Discussion paper	FCNs	Ireland	Clinical practice and Outcomes	Conditions such as faecal incontinence, although clinically less severe than other conditions, generate anxiety, embarrassment and insecurity in sufferers. Communication is essential for the creation of the nurse-patient relationship and the provision of quality services. In addition to communication, consultation and collaboration with patients and their families, nurses must develop skills in interpersonal relationships to be used also within multidisciplinary teams. For this, an educational program is very important.

40	Kent S. (2011)	Community nurses' child protection role: views of public health nurses in Ireland	To present the results of a qualitative analysis who explored the views of a group of public health nurses regarding their role with pre-schoolers	Qualitative study	FCNs	Ireland	Clinical practice	The activities of the FCN also include the protection and care of children. The role of the public health nurse in child protection concerns the prevention of child abuse, relationships with social workers and the ability to identify households at risk. The study reveals that many nurses detect situations of potential risk but are unable to manage them adequately. More training is needed in this regard.
41	Kwok T. (2008)	A randomized controlled trial of a FCN-supported hospital discharge programme in older patients with chronic heart failure	To evaluate the effectiveness and cost-effectiveness of a FCN-supported hospital discharge program in preventing readmissions, in the improvement of the functional status and handicap of older patients with chronic heart failure	A randomized controlled study	Elderly patients	China	Clinical practice and Outcomes	The study shows that there is no significant difference between the results of the control group and that followed by the FCNs in terms of readmission rates ($p = 0.233$) and the 6-minute walking test; Significant results were instead recorded in favour of the intervention group with regard to autonomy and the average of re-hospitalizations ($p = 0.057$).
42	Lalani M. (2019)	Transforming FCN services in the UK; lessons from a participatory evaluation of the implementation of a new community nursing model in East London based on the principles of the Dutch Buurtzorg model	To explore the extent to which some of the principles of the Buurtzorg model could be adapted to FCN	Qualitative study	FCNs	UK	Organizational and educational models	Although the Burtorzg model was born in the Netherlands in a health, social, financial and cultural context very different from that of the United Kingdom, the study notes positive outcomes regarding the patient's care experience, understood as better access to and continuity of care. t
43	Lee G. (2017)	The future of community nursing: Hospital in the Home	To describe the "Hospital in the home" service as a useful service for aged people	Discussion paper	A 78 aged women with COPD riacutization	UK	Organizational and educational models	The case study examines a community care model called "The hospital at home". The case study highlights the ability to assess, treat and manage a patient with an acute event in the community without the need for hospitalization. This type of integrated

								multidisciplinary care model is a valid alternative to traditional care models in both acute and community settings. The case study demonstrates the model's ability to deliver a comprehensive package of multidisciplinary care in a person's home.
44	Looman W.S: (2020)	Teaching Systems Thinking for Advanced Family Nursing Practice: A Theory-Based Tool	Evaluate the effect of use of The Family Nursing Assessment and Intervention Map (FN-AIM) as an educational tool to support student skill development as part of a graduate family nursing course in the United States	Discussion paper	Nursing students	USA	Organizational and educational models and Advanced training program	The study examines a training model, the "The Family Nursing Assessment and Intervention Map" (FN-AIM). The FN-AIM is a pedagogical tool based on the structure of family systems and consists of 7 fields. The course is structured around learning objectives that embrace the cognitive and affective domains of learning, with assignments that mirror the indicators of systemic thinking. Students demonstrated a better approach after using the FN-AIM mapping system.
45	Lumbers M. (2021)	Osteomyelitis, diabetic foot ulcers and the role of the FCN	Describe the epidemiology, characteristics and management of diabetic foot and infection-related and the role of nurses in his management	Discussion paper	FCNs	UK	Clinical practice	FCN plays an important role in the treatment of chronic diseases, including the diabetic foot. This role is supported by local and national guidelines, protocols and personal training. In the management of these patients, the educational activity and the assumption of both the patient and the family are fundamental.
46	Ma W. (2018)	Roles and activities of FCNs in China: a descriptive study	To collect information about the current status of FCNs in China; and explore the roles and activities that they perform in their daily community setting	Cross sectional study	FCNs	China	Clinical practice	The study notes that the daily routine of FCNs most frequently involved vaccinations and primary care services, while the main roles were those of caregiver, educator, organizer and manager.
47	MacDonald J.M. (2005)	Combination model of care for FCN practitioners	Analyse and discuss the combined model and establish whether or not the FCNs were able to achieve a higher level of practice.	Qualitative study	Nurse practitioner consultations and nurses	UK	Organizational and educational models	The study shows a new theory for a higher level of clinical practice, following the HADPIPE model which aimed to investigate: History of the problem, Evaluation of patient problems, Diagnosis of disease,

					based in ambulatory hospital settings			Implementation of care, Discharge of the patient, Evaluation of treatment.
48	Marcadelli S. (2019)	Policy proposals for a new welfare: the development of the family and FCN in Italy as the key to promote social capital and social innovation	To discuss the development of the family and community health care in Italy by focusing on three levels organizational, political and theoretical	Mixed method study	Nurses and Stakeholders	Italy	Clinical practice and core competencies	FCN has an important social value as part of the multidisciplinary team and ensuring a continuity of care because of his involvement in the creation of social support for community's needs and for the prevention and treatment of the chronic diseases as a quality for the improvement in nursing care.
49	Martínez-Riera J.R. (2019)	Family and community nursing, chronology of a specialty	Describe the development of community nursing from 1987 to 2018	Cross sectional study	FCNs	Spain	Clinical practice	FCN is prioritized, as part of a development of the nursing role, because able to act for the primary and community care.
50	McCrae N. (2014)	The role of FCNs in preventing female genital mutilation (FGM)	Explain the context and culture within which the FGM originates and the ways in which FCNs can recognize and deal with situations at risk	Discussion paper	Women and girls who are victims of female genital mutilation	UK	Clinical practice	FCN has a duty in recognizing women and girls at risk of FGM and supporting them before and after the FGM
51	McKenzie J.E. (2007)	A sense of security for cancer patients at home: the role of FCNs	Expose the presently unrecognized minutiae of FCNs' work with cancer patients at home, and to identify the ways in which these, combined to form comprehensive care episodes, contribute to physical and psychosocial well-being.	Qualitative study	Patients and FCNs	Australia	Outcomes	Visit at home from registered nurses are fundamental to strengthen the involvement of patients and their families in the educational programms. This allow patients the possibility to be followed at home even if being re-admitted to hospital.
52	Mendes A. (2017)	A FCN's role in supporting a 'good' experience of grief	Describe the main aspects of grief and the skills nurses need to support the family and the patient	Discussion paper	Dying patient	UK	Clinical practice	FCNs can and do make a difference to better support patients dying and even before that difficult moment for him and his family.
53	Mendes A. (2018)	Personal beliefs, culture and religion in	To help FCNs to raise awareness of their own values and those	Discussion paper	Ethical dilemmas	UK	Core competencies	It's important for FCNs taking care of patients without judgment, working hard to

		community nursing care	of the people they care for to better address the ethical dilemmas that may be encountered		within health care			deliver impartial and individualized care with the utmost compassion and sensitivity.
54	Mnisi S.D. (2012)	Role of FCNs in the prevention of tuberculosis in the Tshwane Health District of Gauteng	To describe the role of FCNs in the prevention of tuberculosis (TB) in order to improve the part they play in the comprehensive management of TB patients; and to identify problems experienced by them when fulfilling this role.	Cross sectional study	Registered Nurses	South Africa	Clinical practice	FCNs has a key function in educating patients in high-risk areas for that infections, such as TB, which is still spreading within the community.
55	Morin D. (2009)	From hospital to home after cardiac surgery	To evaluate the implementation process of FCN care management model; to assess the effect of this model on patients followed at home.	Quasi-experimental study	Hospitalized patients	Canada	Organizational and educational models	The influence of the FCN for the Care Management Model encouraged reorganization and harmonization of nursing practices for patients' cardiac surgery post-intervention following them even after discharge from hospital.
56	Nazarko L. (2013)	Cognitive assessment: a guide for FCNs	Provide a brief explanation of common screening tools and explains the FCN's role in identifying people who may have undiagnosed dementia	Discussion paper	Patients with dementia	UK	Clinical practice	FCN can improve diagnosis rates and ensure the adequate patient's assess to treatment.
57	Nazarko L. (2016)	Considering the difficulties with dysphagia: an overview for FCNs	Describe dysphagia, its impact on sufferers and the role of the FCN in this regard.	Discussion paper	Patients with dysphagia	UK	Clinical practice	FCN has to support patients and their families for the management of dysphagia which could determine clinical problems in elderly.
58	Nissanholtz–Gannot R. (2020)	FCNs and chronic disease in Israel: Professional dominance as a social justice issue.	To analyse comparative changes in FCNs' roles in Israel in recent years, especially related to chronic disease, which can underlie health inequalities	Qualitative study	55 health system professionals	Israel	Clinical practice	FCNs role has expanded a lot, ensuring an improvement of chronic diseases' management and a high-level care.
59	Nissanholtz-Gannot	The changing roles of FCNs: the case of health	To identify recent changes in the roles of health plan nurses and their current areas of	Qualitative study	55 senior nursing and	Israel	Outcomes	FCNs had referred high level of work satisfaction, which is possible for a holistic patient care a delivery care continuity.

	R. (2017)	plan nurses in Israel	activity. It also explored the experience of front-line nurses with regard to autonomy, work satisfaction, and barriers to further role development.		medical professional; 692 FCNs			They felt an improvement in professional autonomy.
60	Norman K.M. (2015)	The image of community nursing: implications for future student nurse recruitment	The article reports the results of a research that investigated the opinions of 40 young people regarding nursing as a profession	Qualitative study	Nursing students from four schools in the West Midlands	UK	Core competencies	The holistic vision of patients and their caregiver allow the knowledge, skills and leadership capabilities to ensure an adequate planning and decision-making strategies
61	Ogston-Tuck S.A. (2018)	A silent epidemic: community nursing and effective pain management	To dispel the myths and improve clinical practice, particularly in effective pain assessment.	Discussion paper	Patients with chronic pain	UK	Clinical practice	The FCN's pain assessment is fundamental, in particular for the recognition of factors that can influence one's experience of pain and it is fundamental for the approach to pain management.
62	Oliver S. (2009)	Understanding the needs of older people with rheumatoid arthritis: the role of the FCN	This article outlines the particular challenges associated with rheumatoid arthritis in older people and how FCNs can contribute to assessing patients and helping to meet their needs for care.	Discussion paper	Older patients	UK	Clinical practice	FCNs have a vital role in supporting older people with rheumatoid arthritis in symptom and disease control
63	Omeri A. (2004)	Cultural diversity: A challenge for FCNs	To support FCN to provide nursing care congruent with the different lifestyles and customs found in the community	Discussion paper	Different cultural groups with distinctive features of culture, ethnicity and disability	Australia	Core competencies	FCNs still struggle to approach different types of patients with different clinical situations. Nurses acting within the community is fundamental to achieve safe care.
64	Østergaard B. (2021)	Effect of family nursing therapeutic conversations on	Evaluate the short-term, medium-term and long-term effect of family nursing	Randomised control study	Patients with heart	Denmark	Outcomes	Patients and their families feel more supported both at short-term (p = 0.002) medium-term (p = 0.008) and long-term (p =

		patients with heart failure and their family members: secondary outcomes of a randomised multicentre trial	therapeutic conversations added to conventional care versus conventional care on social support, family health and family functioning in outpatients with heart failure and their family		failure clinics			0.018) among patients and their family members (p = <0.001; 0.007 and 0.014 respectively) in the intervention group in comparison with the control group. They reported an increased reinforcement, feedback, decision-making capability and collaboration with the nurse.
65	Papado poulou . (2021)	Perceptions, practices and educational needs of FCNs to manage frailty	To understand nurses' perceptions of frailty in a community setting and their needs for education on its assessment and management	Qualitat ive study	FCNs	UK	Advanc ed training progra m	FCNs identified specific education about frailty patients and suggested to introduce this topic in undergraduate and postgraduate nursing programmes.
66	Papatha nasiou I. (2020)	Family Community Nursing Curriculum	To submit a Curriculum for the Training of FCNs	Project	FCNs facing 1 year of learning program me	Greece	Advanc ed training progra m	Training programme, based on the ENhaNCE FCN reference curriculum, consists of 10 modules for a totlae of 250 hours of teaching and 1000 of practical internship.
67	Phelan A. (2010)	Elder abuse and the FCN: supporting the patient	To explain what elder abuse is, consider the FCN's role in elder abuse and describe details practice guidelines and challenges related to the issue	Discussi on paper	Elderly patients	UK	Clinical practice	The prevention of elderly abuse, which is one of the many areas of FCNs' caring is fundamental for that nurses who acts within patient's caregivers and homes.
68	Phillips A. (2016)	Supporting smoking cessation in older patients: a continuing challenge for FCNs	To discuss how FCNs can support their older patients to quit smoking by fostering a patient-centered partnership through good communication and empathy.	Discussi on paper	Older smoking patients	UK	Clinical practice and core compet encies	Communication strategies are fundamental a patient centered-care as part of that competences which guide patients' in smoking cessation, as part of the specific caring areas of FCNs.
69	Pickstoc k S.(2017)	Breathlessness at end of life: what FCNs should know	To support FCNs to gain knowledge patients and their families to manage breathlessness at end of life	Discussi on paper	Dying patients	UK	Clinical practice	FCN has the fundamental role in knowing the history of breathlessness to identify and managing reversible pathologies.
70	Pisano Gonzále z M.M. (2019)	Community and family nurse: Present keys, future challenges	Describe the stages of development of training for FCNs in Spain, the results obtained and future scenarios	Discussi on paper	Commu nity nursing training	Spain	Core compet encies and Advanc ed training	Graduate training and learning was fundamental for FCNs to acquire more competencies for families and the entire community.

							program	
71	Randall D. (2021)	Community nursing: will new standards be a panacea?	To show how, in past, the FCN, has attempted to create an economy of scale by merging community nursing with public health work without meeting the needs of nurses	Discussion paper	Nursing and Midwifery Council's standards for specialist practice	UK	Advanced training program	An urgent need for SPQ (Specialist Programme Qualification) courses has been recognized by nurses.
72	Reid J. (2014)	Managing urinary incontinence: guidelines for FCNs	To outline basic bladder function, detail the causes and types of urinary incontinence, and explore the role of the FCN in assessment and management.	Discussion paper	Patients with urinary incontinence	UK	Clinical practice	FNC's activity is the one that could allow the recognition of symptoms, analysis of the person's lifestyle, knowledge of medications, patient support and management.
73	Roden J. (2016)	Australian rural, remote and urban FCNs' health promotion role and function.	To investigate to sustainability of the health promotion activity carried out by Australian FCNs	A mixed-methods study	Rural FCNs and of Urban FCNs	Australia	Clinical practice and Outcomes	Urban FCNs had a narrower view on caring for individuals rather than group Rural nurses had more positive attitudes towards health promotion.
74	Sasso L. (2018)	Report on current FCN working and occupational contexts	To provide a overview of current work and employment contexts of FCN	Discussion paper	21 countries	Europe	Core competencies	FCN must be able to adapt care to the needs of the individual. To do this, it must have skills and knowledge and be based on scientific evidence and best practices.
75	Savini S. (2021)	A family nurse-led intervention for reducing health services' utilization in individuals with chronic disease: the ADVICE pilot study	To assess whether a structured family nurse-led educational intervention was effective in reducing disease-related hospital readmissions. 2. To investigate the factors associated with the risk of health services' use.	Quasi-experimental study	Patients	Italy	Clinical practice and Outcomes	Educational intervention consisted of face-to-face sessions, fundamental for changeset in behavior and this let FCNs feel satisfied about their job.
76	Simonetti V. (2021)	Effectiveness of family nurse-led programme on accuracy of blood pressure self-management: a randomised controlled trial	to evaluate the effectiveness of a Family Nurse Practitioner (FNP)-led programme on the degree of adherence of current recommendations on home blood pressure	A randomized control study	Patients	Italy	Clinical practice and Outcomes	Almost 75% of the patients in the intervention group,, after FNP intervention, demonstrated a better adherence of blood pressure measurement.

			self-management (HBPM) as compared to routine care and management.					
77	Skingley A. (2016)	Older people, isolation and loneliness: implications for community nursing	Defining social isolation and loneliness and describe the implication for community nursing	Discussion paper	Older patients	UK	Clinical practice	FCNs have to consider the entire social condition of patients, minimizing loneliness and social isolation.
78	Slevin E. (2003)	Learning disabilities: a survey of FCNs for people with prevalence of challenging behavior and contact demands	To identify the overall caseload sized of the nurses, the prevalence of people with learning disabilities. 2. To discover courses or training that helped the nurses to fulfil their roles.	Cross sectional study	65 patients with learning disabilities	UK	Clinical practice	The CNLD has been defined as a special FCN as a member of the primary health care team who provides specialist advice to clients, their families and carers and to other professionals'.
79	Stuart E. (2020)	Nutrition and wound care: what FCNs should know	Wound healing: often coming at a great cost to patient quality of life, as well as the undeniable significant financial cost to the NHS.	Discussion paper	Patients with leg ulcers	UK	Clinical practice	FCNs are part of the multidisciplinary team for improve healing as the fundamental part of the nutritional assessment.
80	Terracciano E.(2020)	The effect of FCN on mortality and hospitalization in a group of over-75 older adults: a nested case-control study	To explore the causal association through the analysis of the hospitalization and mortality rate after a pro-active social service integrated by the FCN	Cross sectional study	Groups of patients having access to different kind of services.	Italy	Outcomes	A lower re-admission in patients was showed after a pro-active social service integrated by the FCN, if compared to the control group.
81	Vogel R.G. (2021)	Process evaluation of a program to empower FCN leadership	To describe the process evaluation of NitL (Nurses in the Lead)	Mixed method study	97 FCNs and 31 team members,	Netherland	Outcomes	NitL programme led patients' and their families feel involved in the educational programme, led the FCN feel useful in their educational role.
82	Wacharasin C. (2008)	Family nursing practice, education, and research. What is happening in Thailand?	To describe the evolution of FCN in Thailand over the past 20 years	Discussion paper	FCNs	Thailand	Core competencies	The collaboration with the multidisciplinary team is fundamental in education role as well as in the research role of FCNs to provide a safe care.
83	Wang S. (2019)	Improving the curriculum for a community nursing training program in Guangzhou City, China	Improving the curriculum of community nursing training in Guangzhou	Cross sectional study	318 participating nurses and instructors and	China	Clinical practice and Advanced training	Nurses identified as priorities DT2M management, breast-self-check for public health. Furthermore, the final curriculum had 10 modules including 27 courses and 117

					22 experts		program	items, based on the FCNs Training Outline.
84	Weber S. (2010)	A stigma identification: framework for family nurses working with parents who are lesbian, gay, bisexual or transgendered and their families	Identify what is stigma and how can family nurses be helped to better understand the nature of stigma.	Discussion paper	FCNs	USA	Clinical practice	FCNs can have a positive impact for stigma and social marginalization on the mental health on LGBT patients and can counteract this stigma in positive ways.
85	Widyarani D. (2020)	Identifying Community/Public Health Nursing Competencies in Indonesia: A Modified Delphi Method	To identify the competencies of the Indonesian nurses from experts using the C/PHN competencies of the Quad Council Coalition.	Cross sectional study	8 experts from three universities with expertise of public health nursing and hold a master degree in nursing.	Indonesia	Core competencies	Communication skills are defined as one of the competencies of FCNs.
86	Wilkes L. (2014)	Clients with chronic conditions: FCN role in a multidisciplinary team	To define and validate the role of the FCN in a multidisciplinary team that takes care of peers with chronic and complex needs	Cross sectional study	FCNs	UK	Core competencies	The role of the FCN is fundamental in a multidisciplinary team and has six main domains: defender, advocate, coordinator, educator, team member and evaluate.
87	Wood-Baker R. (2012)	Clinical trial of FCN mentoring to improve self-management in patients with chronic obstructive pulmonary disease	To investigate the effect of a program increase self-management behaviours delivered by FCNs, compared with usual care, on health-related quality of life and health care utilization in people with COPD following hospitalization.	Randomized control study	Patients with acute exacerbation of COPD	Australia	Outcomes	FCNs has a fundamental role in reducing hospital re-admissions in exacerbation of COPD.
88	Yates A. (2019)	Basic continence assessment: what	Describe incontinence, its impact on sufferers	Discussion paper	Patients with incontin	UK	Clinical practice	FCNs promote health even in patients with incontinence disorders. Their role is

		FCNs should know	and the role of the FCN in this regard.		ence disorder			fundamental to improve patients' quality of life.
89	Yingling C.T. (2017)	Building nurses' capacity to address health inequities: incorporating lesbian, gay, bisexual and transgender health content in a family nurse practitioner programme	To describe the experience of incorporating LGBT pulping health content into the curriculum of FCN at a nursing college in the United States	Discussion paper	Community nursing students	USA	Advanced training program	The experience incorporating LGBT-specific content into the FCN program has proven positive for community nursing students.
90	Zimansky M. (2020)	Effects of implementing a brief family nursing intervention with hospitalized oncology patients and their families in Germany: a quasi-experimental study	To investigate the effects of implementing family nursing care on several psychological and physical outcomes of patients and their family members	Quasi-experimental study	Patients and family members	Germany	Outcomes	FCNs are the figures involved into the psychological support of cancer patients.
